# aBEAT: A Toolbox for Consistent Analysis of Longitudinal Adult Brain MRI

**DOI:** 10.1371/journal.pone.0060344

**Published:** 2013-04-03

**Authors:** Yakang Dai, Yaping Wang, Li Wang, Guorong Wu, Feng Shi, Dinggang Shen, Alzheimer’s Disease Neuroimaging Initiative

**Affiliations:** 1 IDEA Lab, Department of Radiology and BRIC, University of North Carolina at Chapel Hill, Chapel Hill, North Carolina, United States of America; 2 Medical Imaging Department, Suzhou Institute of Biomedical Engineering and Technology, Chinese Academy of Sciences, Suzhou, Jiangsu, China; Beijing Normal University, China

## Abstract

Longitudinal brain image analysis is critical for revealing subtle but complex structural and functional changes of brain during aging or in neurodevelopmental disease. However, even with the rapid increase of clinical research and trials, a software toolbox dedicated for longitudinal image analysis is still lacking publicly. To cater for this increasing need, we have developed a dedicated 4D Adult Brain Extraction and Analysis Toolbox (aBEAT) to provide robust and accurate analysis of the longitudinal adult brain MR images. Specially, a group of image processing tools were integrated into aBEAT, including 4D brain extraction, 4D tissue segmentation, and 4D brain labeling. First, a 4D deformable-surface-based brain extraction algorithm, which can deform serial brain surfaces simultaneously under temporal smoothness constraint, was developed for consistent brain extraction. Second, a level-sets-based 4D tissue segmentation algorithm that incorporates local intensity distribution, spatial cortical-thickness constraint, and temporal cortical-thickness consistency was also included in aBEAT for consistent brain tissue segmentation. Third, a longitudinal groupwise image registration framework was further integrated into aBEAT for consistent ROI labeling by simultaneously warping a pre-labeled brain atlas to the longitudinal brain images. The performance of aBEAT has been extensively evaluated on a large number of longitudinal MR T1 images which include normal and dementia subjects, achieving very promising results. A Linux-based standalone package of aBEAT is now freely available at http://www.nitrc.org/projects/abeat.

## Introduction

Brain structure and function change as a result of aging or brain diseases such as Alzheimer’s disease [1]. Magnetic resonance imaging (MRI) provides a safe way to image brain structure and function *in vivo*. Thus, longitudinal MRI is widely used to reveal brain changes in basic and clinical neuroscience studies. For example, Chetelat *et al.* [2] used a longitudinal voxel-based method to map the progression of gray matter (GM) loss in mild cognitive impairment (MCI) patients over time, and found a significant GM loss in brain areas such as temporal cortex and parietal cortex. Nakamura *et al.* [3] further found longitudinal neocortical GM volume reduction in the first-episode schizophrenia, but increase in the first-episode affective psychosis. In addition to these volumetric studies, longitudinal cortical surface change associated with normal aging was also studied in [4] by reconstructing cortical surfaces from longitudinal MR images. They found widespread aging-related cortical thickness decline, especially in frontal and parietal regions [4]. On the other hand, 4D cortical thickness measurement was also developed for studying Alzheimer’s disease (AD) in [5], [6].

Since brain change pattern could be subtle and complicated during aging or in brain diseases, it is important to develop accurate longitudinal analysis tools. To do this, current analysis tools are generally based on independent processing of each time-point image of the same subject, involving the steps of image preprocessing, brain extraction, tissue segmentation, and brain labeling. Specifically, image preprocessing is first used for bias correction and histogram matching for each original MR image. Brain extraction is then used to remove non-brain tissues, such as scalp, skull, and dura [7], while keeping all brain tissues such as white matter (WM), gray matter (GM), and cerebral spinal fluid (CSF). Tissue segmentation is further performed to classify the brain-extracted image into WM, GM, and CSF, which will allow the measurement of overall brain tissue changes over the time. Finally, brain labeling is applied to delineating brain ROIs in each time-point image, which allows the study of longitudinal change of each ROI [8], [9].

Various toolboxes have been developed for this purpose, including ITK [10], FSL [11], FreeSurfer [12], and SPM [13]. However, these toolboxes are mainly developed for analysis of single-time-point images, not for longitudinal images, except FreeSurfer that includes a longitudinal surface reconstruction component. Since brain changes are subtle during aging and in most degenerative diseases [1], especially for a typical longitudinal follow-up of only one to two years [9], [14], it is expected that the analysis results in each step of brain extraction, tissue segmentation, and ROI labeling should be accurate and consistent for the longitudinal images. However, it is challenging for the conventional single-time-point based analysis methods to achieve the longitudinal consistent results, since no temporal guidance is applied.

To address this limitation, we have developed a dedicated 4D Adult Brain Extraction and Analysis Toolbox (aBEAT). Specially, aBEAT provides functions of 4D brain extraction, 4D tissue segmentation, and 4D brain labeling for achieving the consistency in analyzing longitudinal brain MR images. It is worth noting that single-time-point image can be considered as a special case of longitudinal images and thus can also be analyzed by aBEAT. The functions of 4D brain extraction, 4D tissue segmentation, and 4D ROI labeling are provided by the following three 4D image analysis algorithms, respectively:

1: 4D deformable-surface-based brain extraction.

Classic brain extraction algorithms such as BSE [15], BET [16], and graph cut [17] generally perform a single run of brain extraction on a given image. Recently, advanced algorithms were developed to perform multiple brain extractions with multiple atlases or algorithms [7], [18]–[20] and then fuse all results to produce the final result with improved accuracy. However, all these algorithms are not able to achieve consistent brain extraction results from the longitudinal brain images, due to separate extraction of each time-point brain image. To address this issue, we use a 4D brain extraction algorithm [21], which was extended from a 3D deformable-surface-based brain extraction method [22]), for achieving consistent brain extraction results. It is performed by first constructing the initial common brain surface from the group mean of all aligned longitudinal images and then deforming it simultaneously to each time point with the constraint of temporal smoothness.

2: 4D tissue segmentation with cortical-thickness constraint [23].

A number of automated tissue segmentation algorithms [12], [13], [24] have been proposed to segment WM, GM, and CSF from the brain image. However, most of them were designed to segment 3D image. In contrast, CLASSIC [25] was specially designed for simultaneous segmentation of longitudinal brain images using voxel-wise tissue classification framework. However, it still cannot guarantee the consistency of cortical thickness measured on the longitudinal images, which could seriously affect the power of longitudinal study. To address this issue, we incorporate a 4D tissue segmentation algorithm with cortical-thickness constraint [23] into our toolbox. In this algorithm, a 3D coupled-level-sets method [26] is first used to obtain the initial segmentation of WM, GM, and CSF at each time-point, and then a longitudinal cortical-thickness constraint is further used to ensure its temporal consistency during the 4D tissue segmentation.

3: 4D ROI labeling with longitudinal groupwise image registration [27].

Although many pairwise image registration methods (such as Demons [28] and HAMMER [29]) can be used for atlas-based brain labeling, their labeling results for the longitudinal images could be inconsistent, since each time-point image is labeled independently. We thus propose to label all longitudinal images simultaneously with our longitudinal groupwise image registration algorithm [27], which can not only register all longitudinal images jointly to the common space, but also maintain their temporal coherence. Specifically, we will first adopt this algorithm to align all longitudinal images onto a common space for obtaining their group-mean image. Then, we use our symmetric feature-based pairwise registration method [30] to register an atlas with pre-labeled ROIs to this group-mean image. Finally, by combining the respective deformations, we can label the ROIs for each time-point image. Since the temporal coherence is well respected in our method, we will be able to get consistent labeling for different time points.

The performance of aBEAT has been extensively evaluated with a large number of longitudinal brain MR images from ADNI database. Compared with other existing algorithms for brain extraction (e.g., using 3D deformable-surface-based method) and tissue segmentation (e.g., using CLASSIC), aBEAT can achieve superior accuracy and consistency for longitudinal images. Moreover, our brain labeling module in aBEAT also shows promising results for longitudinal images. The remainder of this paper is organized as follows. The methodological description of aBEAT is provided in Section 2. Representative results by aBEAT are demonstrated in Section 3. Finally, discussion is presented in Section 4.

## Methods

### 1. ADNI Database

Data used in the preparation of this article were obtained from the Alzheimer’s Disease Neuroimaging Initiative (ADNI) database (http://adni.loni.ucla.edu). The ADNI was launched in 2003 by the National Institute on Aging (NIA), the National Institute of Biomedical Imaging and Bioengineering (NIBIB), the Food and Drug Administration (FDA), private pharmaceutical companies and non-profit organizations, as a $60 million, 5-year public-private partnership. The primary goal of ADNI has been to test whether serial magnetic resonance imaging (MRI), positron emission tomography (PET), other biological markers, and clinical and neuropsychological assessment can be combined to measure the progression of mild cognitive impairment (MCI) and early Alzheimer’s disease (AD). Determination of sensitive and specific markers of very early AD progression is intended to aid researchers and clinicians to develop new treatments and monitor their effectiveness, as well as lessen the time and cost of clinical trials.

The Principal Investigator of this initiative is Michael W. Weiner, MD, VA Medical Center and University of California–San Francisco. ADNI is the result of efforts of many co-investigators from a broad range of academic institutions and private corporations, and subjects have been recruited from over 50 sites across the U.S. and Canada. The initial goal of ADNI was to recruit 800 adults, ages 55 to 90, to participate in the research, approximately 200 cognitively normal older individuals to be followed for 3 years, 400 people with MCI to be followed for 3 years and 200 people with early AD to be followed for 2 years.” For up-to-date information, please see www.adni-info.org.

### 2. Overview of aBEAT

**Figure 1:**
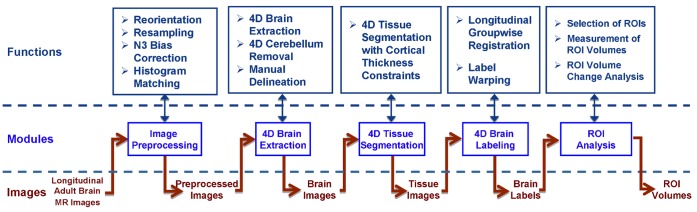
The architecture of aBEAT. The user is free to process data using either an individual module or the entire pipeline (from image preprocessing to ROI analysis).

The architecture of aBEAT is shown in Fig. 1. The complete data processing pipeline consists of five major modules (see the five blue boxes in the middle row of Fig. 1). Briefly, the image preprocessing module normalizes the original images and corrects their intensities. The 4D brain extraction module consistently removes non-brain tissues (such as scalp and skull) and keeps brain tissues (including WM, GM, and CSF) from the preprocessed longitudinal images of each subject. The serial brain tissues of each subject are then jointly segmented by the 4D tissue segmentation module. Next, the 4D brain labeling module simultaneously warps an atlas with pre-labeled ROIs onto the longitudinal images for ROI labeling. Finally, longitudinal ROI volumes and the volume changes for all subjects can be automatically measured and displayed using the ROI analysis module. Major functions in each module are also listed in the top row of Fig. 1. It’s worth noting that the processing pipeline of the architecture is similar to that of our previously developed toolbox iBEAT [31]. However, the iBEAT is a dedicated toolbox for analysis of infant brain MR images, which have poor image quality, low tissue contrast, and most importantly the dynamic tissue change over time. Thus, all steps used for infant brain extraction, tissue segmentation, and brain labeling are different from the adult brain analysis, and definitely much different from the longitudinal image analysis. On the other hand, the 4D processing algorithms integrated in each functional module of aBEAT are specialized for the consistent analysis of longitudinal adult brain MR images, and are thus completely different from the processing algorithms in iBEAT. In addition, there is no ROI analysis module in iBEAT.

**Figure 2:**
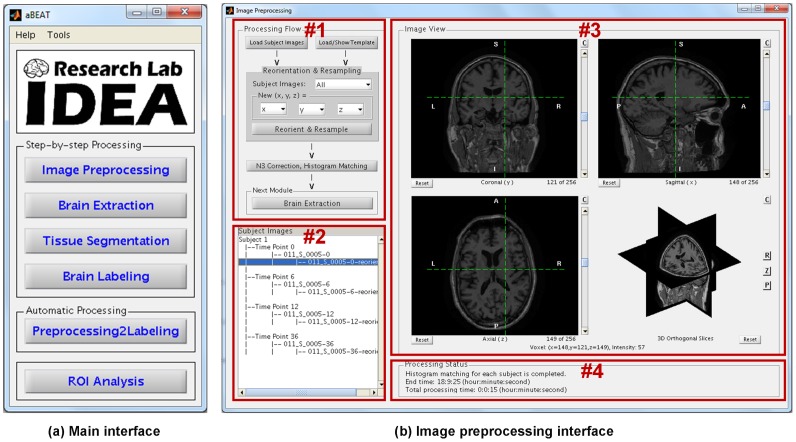
The main interface and image preprocessing interface in aBEAT. (a) The main interface includes the menus for activating all five major processing modules (refer to Fig. 1). In addition to step-by-step processing, input images can be processed automatically from image preprocessing to brain labeling. (b) The upper left panel (#1) displays step-by-step functions for image preprocessing. The bottom left panel (#2) lists the input images and generated images. The upper right panel (#3) displays a selected image. The bottom right panel (#4) shows the image processing status. The interfaces of other modules are similar to the interface of this preprocessing module, except for the functions listed in the processing flow panel (#1).

Parallel computing strategy is used in aBEAT for fast processing. Specifically, each image is processed by a thread in the image preprocessing module, while in the 4D modules such as brain extraction, tissue segmentation, and brain labeling, each subject is processed by a thread. It is worth noting that the current computer generally has multiple CPU cores, thus the use of the parallel computing strategy can largely reduce the computation time. The graphical user interfaces (GUIs) and the overall framework of aBEAT were implemented in MATLAB, while the modules and functions in aBEAT were implemented with the combination of C/C++, MATLAB, Perl and Shell scripts. The main interface and image preprocessing interface in aBEAT are shown in Fig. 2. Specifically, the main interface (see Fig. 2(a)) includes the menus for activating all five major processing modules (refer to Fig. 1). In addition to the step-by-step processing, the input images can be processed automatically from image preprocessing to brain labeling. The image preprocessing interface (see Fig. 2(b)) includes the step-by-step functions for image preprocessing. The interfaces of other modules are similar to the interface of this preprocessing module, except for the functions listed in the processing flow panel (#1).

### 3. Image Preprocessing

**Figure 3:**
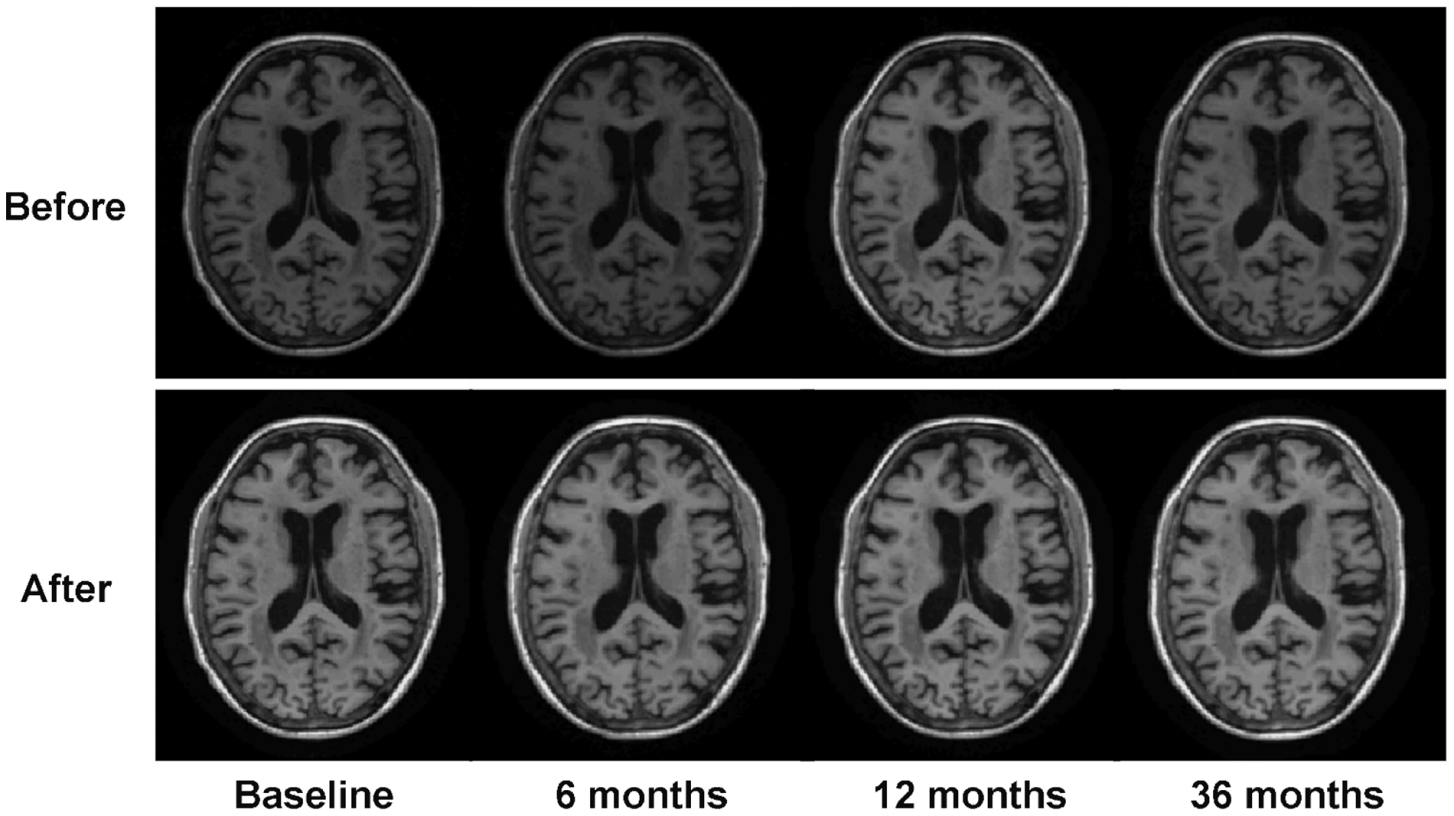
Illustration of N3 correction and histogram matching on serial images of one subject at 4 time points. Axial slices of the serial images before and after the processing are shown, respectively. We can see that the intensity inhomogeneity and inconsistency of the serial images are removed clearly.

Since the orientations, voxel sizes, and volume sizes of original input images may be different, aBEAT first reorients and resamples each image to a standard format, for facilitating further data analysis. Specifically, the standard orientation of aBEAT follows the RAS (Right, Anterior, and Superior) coordinate, which is a standard neurological convention and widely used in other neuroimaging software such as MRIcro [32], SPM [13], and eConnectome [33]. The standard voxel size and volume size in aBEAT are set as 1×1×1 mm^3^ and 256×256×256, respectively. The input images, whose original orientations are not in the RAS coordinate, are reoriented semi-automatically. Specifically, first the input image is reoriented tentatively with all valid reorientation parameters (obeying the right-hand rule). Then, the user can check all tentatively-reoriented images in the GUI and determine the right one that matches with the RAS coordinate system. Using the right reorientation parameters, aBEAT can reorient the input image, as well as other images that have the same original orientation, into the RAS coordinate. After all input images are reoriented and resampled, N3 bias correction [34] is performed on each of these images to remove intensity inhomogeneity. Finally, for each subject, the histograms of follow-up images are matched to the histogram of the baseline image to remove intra-subject intensity variations. Fig. 3 shows the N3 correction and histogram matching result for one subject.

The reorientation, resampling, and N3 bias correction functions were implemented based on the FSL library (Analysis Group, FMRIB, Oxford, UK), ITK toolkit (Kitware Inc.), and MINC package (McConnell Brain Imaging Centre of the Montreal Neurological Institute, McGill University), respectively. In addition to the image preprocessing functions, a variety of functions were also implemented in aBEAT to support interactive inspection of MR images, including display of image slices, mouse-driven image slicing, zooming, translation, and rotation.

### 4. 4D Brain Extraction

A 4D deformable-surface-based brain extraction algorithm [21] was implemented in aBEAT to remove non-brain tissues (such as scalp, skull, and dura) simultaneously from the preprocessed images and produce consistent brain images for the following step of tissue segmentation.

#### 4.1 4D Deformable-surface-based brain extraction

The 4D brain extraction algorithm, which was extended from our 3D deformable-surface-based brain extraction algorithm [22], consists of two steps: initialization of deformable surfaces, and consistent brain extraction with the deformable surfaces.

1: Initialization of Deformable Surfaces.

The initial deformable surfaces that roughly represent the brain boundaries of longitudinal images of a subject are obtained as follows. First, the preprocessed longitudinal brain MR images (with skull) are affine-aligned to their common space using a groupwise affine registration algorithm [35], for avoiding any potential bias due to the selection of template. Second, a brain probability map, attached with the MNI (Montreal Neurological Institute) brain atlas [36], is warped onto the affine-aligned image of each time-point by linear registration via FLIRT [37], followed by nonlinear registration via Demons [28]). Notice that the brain probability map in the space of MNI brain atlas was obtained by aligning and averaging a population of brain MR images with manually-delineated brain masks [22]. Third, the warped brain probability map is used to remove most non-brain voxels (scalp, skull, and dura) for the respective image of each time-point. Fourth, a spherical volume is estimated for each brain-extracted image of each time-point, according to its intensity and spatial distributions of brain voxels (WM, GM, and CSF). Notice that each estimated spherical volume is represented by its center of gravity (COG) and radius. Finally, the averaged COG and radius of all estimated spherical volumes from all brain-extracted images of all time-points are used to construct a common spherical surface, which is then imposed onto each time-point image as the initial brain surface.

2: Consistent Brain Extraction with Deformable Surfaces.

The above-obtained initial brain surfaces for the longitudinal images are deformed to achieve consistent brain extraction, typically with 1000 iterations [16]). Specifically, during the evolution of the deformable surface for each time-point image, four forces are placed at each vertex of the deformable surface to drive the surface deformation, which includes (1) spatial-smoothness force to smooth surface and obtain evenly spacing vertices; (2) image-intensity-based force to separate brain voxels from non-brain voxels; (3) brain-probability-map-guided force to drive the vertices to the true brain boundary; (4) temporal-smoothness force to drive each vertex to the center of its corresponding vertices in the temporal neighbors. Specially, with the temporal-smoothness force, we can obtain more accurate and temporally-consistent brain extraction results for the longitudinal brain images, compared with the case of using the 3D deformable-surface-based brain extraction method [22].

#### 4.2 4D Cerebellum removal and manual delineation

**Figure 4:**
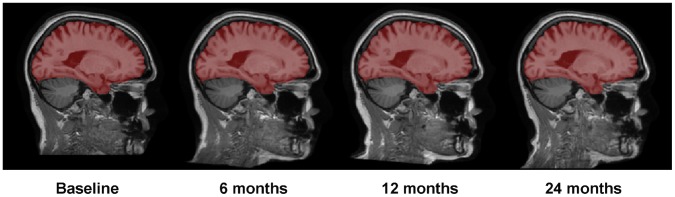
Demonstration of 4D brain extraction and cerebellum removal on four time-point images of a subject. Cerebrums are extracted consistently.

Automatic 4D cerebellum removal is performed based on the above brain extraction result for keeping only the cerebrum in the final image, as detailed below. First, as similarly described above, those brain-extracted longitudinal images are simultaneously registered with a groupwise affine registration algorithm [35], to further obtain their group-mean image. Then, the MNI brain atlas [36] is registered with this group-mean image using FLIRT [37], followed by Demons registration [28]. Finally, the cerebellums in the brain-extracted longitudinal images are simultaneously removed by the warped cerebellum mask from the MNI brain atlas. Fig. 4 shows the 4D brain extraction and cerebellum removal results on the preprocessed images at four time-points for a subject.

If needed, the automatic 4D brain extraction results can be further refined by a manual editor provided in aBEAT. In this manual edition step, a colored brain mask (as shown in Fig. 4), representing the automatically-extracted brain of each time-point, will be overlaid on the corresponding preprocessed brain image. Then, a 3D (or 2D) painter or eraser tool can be used to edit each brain mask interactively in the three orthogonal slices (i.e., axial, coronal, and sagittal). Mouse-driven image inspection functions, such as image slicing, zooming, and translation, are also available in the manual editor for convenient editing. The final edited brain masks can be used to generate the final brain extraction results for the longitudinal images.

### 5. 4D Tissue Segmentation

The 4D tissue segmentation algorithm [23], which integrates local intensity distributions, spatial cortical-thickness constraint, and temporal cortical-thickness consistency constraint into a level-sets framework, was implemented in aBEAT to achieve consistent tissue segmentation for the longitudinal images.

Specifically, three level-set functions are used to separate WM, GM, CSF, and background intensities of each time-point image, where the zero-level surfaces of the level-set functions are the interfaces of WM/GM, GM/CSF, and CSF/background, respectively. Three terms, i.e., data fitting energy, spatial cortical-thickness constraint, and temporal cortical-thickness consistency constraint, are integrated into the level-sets framework. The three terms are briefly described below:

1: The data fitting term integrates local intensity distributions of current image and also the tissue probability from the population data. Specifically, the local intensity distributions are modeled for WM, GM, and CSF, respectively, by using Gaussian distributions with spatially-varying means and covariance matrices.
2: The spatial cortical-thickness constraint is proposed to preserve the cortical thickness (i.e., the distance between the surfaces of WM/GM and GM/CSF) within a biologically reasonable range (i.e., 1∼6.5 mm according to the literature), to guide the surface evolution during the segmentation [38].
3: The temporal cortical-thickness consistency constraint is proposed for consistent cortical segmentation of longitudinal images by making the estimated cortical thickness of current time-point in-between those at the immediate temporal neighbors [23].

**Figure 5:**
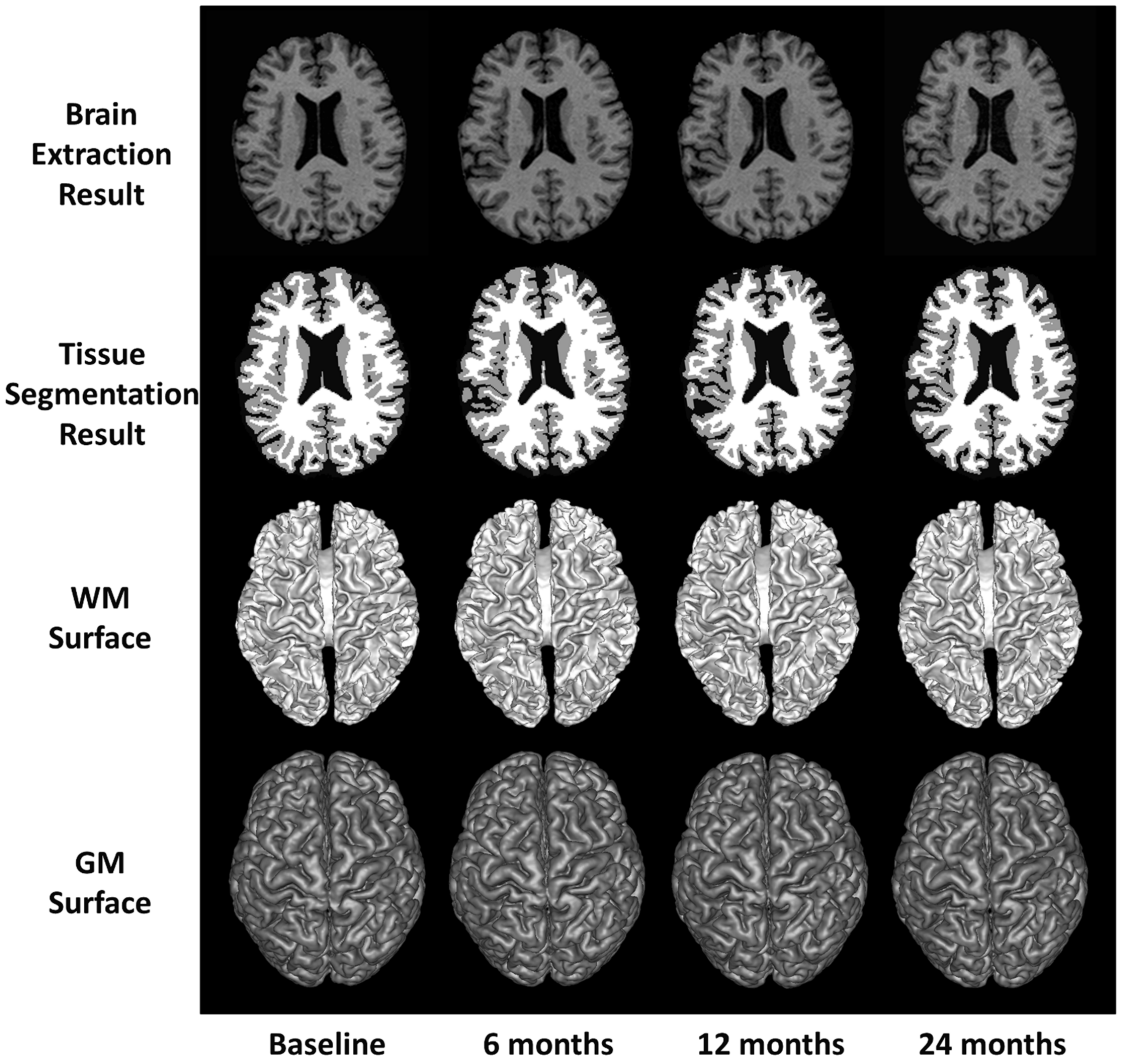
Demonstration of 4D tissue segmentation result. WM, GM, and CSF tissues are segmented from the brain-extracted longitudinal images of a normal control subject at 4 time points. Both WM and GM surfaces are also displayed to show their consistency across different time-points.

The 4D tissue segmentation is then achieved by optimizing the above level-sets framework. First, an initial 3D segmentation using only the data fitting term and the spatial cortical-thickness constraint, also called as coupled level-sets [26], is performed at each time-point separately. Second, 4D registration [39] is performed based on the current segmentation results to obtain the difference of cortical thickness between neighboring time points. Third, the proposed 4D segmentation using data fitting term, cortical-thickness constraint, and temporal cortical-thickness smoothness constraint [23] is performed at each time-point image for joint segmentation. The second and third steps are performed alternately until convergence. It is worth indicating the importance of selecting good initialization for the three level-set functions. We adopted the initialization method in [26], where a convex optimization method was employed for initialization by using both global image statistical information and atlas spatial prior. The related parameters were chosen based on the cross-validation. This method has been proven robust by taking advantage of both global statistics and atlas prior. More details can be referred to [26]. Fig. 5 shows the 4D tissue segmentation result for longitudinal images of a normal control subject.

### 6. 4D Brain Labeling

A novel longitudinal ROI labeling framework was developed in aBEAT to consistently label brain ROIs for the longitudinal images of subject. The MNI brain atlas [36] is used to label each longitudinal image into 45 ROIs in each hemisphere. It is worth noting that customized brain atlases can also be used in aBEAT for brain labeling.

**Figure 6:**
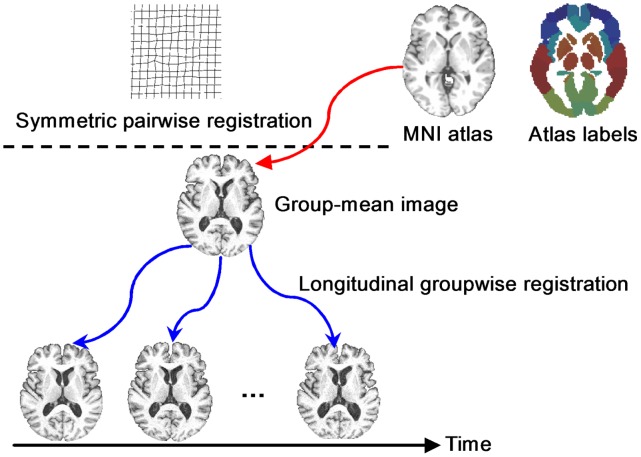
Illustration of our longitudinal ROI labeling framework. The labels in MNI atlas are consistently warped, via the group-mean image, onto all time-point images of the subject.

The general framework of our longitudinal ROI labeling is given in Fig. 6, which consists of two steps. In the first step, all longitudinal images of a subject are simultaneously registered to their group-mean image in the common space by our longitudinal groupwise image registration algorithm [27]. Specifically, we hierarchically select a set of key points with distinctive features to guide the registration between the tentatively-estimated group-mean image (in the middle of Fig. 6) and different time-point images by robust feature matching. Since the key points are located at distinctive regions, their correspondences can be identified more reliably. These key points are used as driving points to steer the whole registration. Meanwhile, by mapping the group-mean image onto the domain of each time point, every key point in the group-mean image has several warped points in different time points, which can be assembled into a time sequence to form a temporal trajectory. Therefore, the temporal coherence within longitudinal images can be assured by deploying kernel smoothing along all these temporal trajectories. Next, thin-plate splines (TPS) are performed to interpolate the dense deformation field for each time-point image, by considering all key points as control points in TPS. Given these tentatively-estimated spatiotemporal deformations, their average deformation will be used to further update the group-mean image. By repeating the above procedure (which includes correspondence detection, kernel smoothing, dense deformation interpolation, and group-mean image updating), we can finally obtain the spatiotemporal deformation fields (blue curves in Fig. 6) of all images to the group-mean image in the common space.

**Figure 7:**
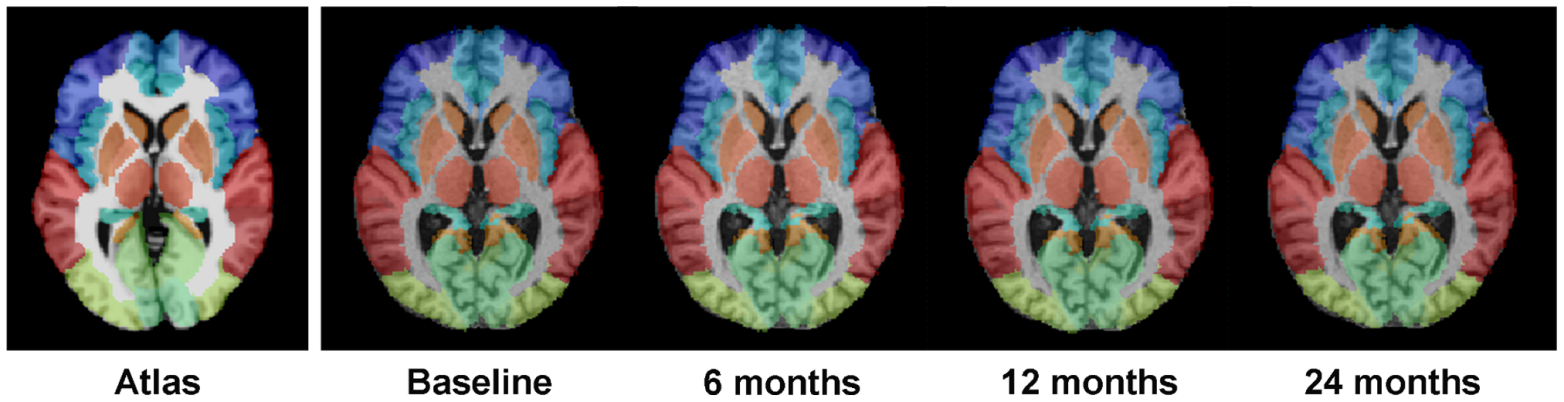
Demonstration of 4D brain labeling result on longitudinal brain images of a normal control subject at four time-points. The MNI brain atlas is shown in the first column, and different ROIs are shown with different colors.

In the second step, a symmetric feature-based pairwise registration [30] is performed to estimate the deformation field (red curve in Fig. 6) from the MNI atlas image to the group-mean image of the subject. Finally, the deformation pathway from each longitudinal image to the MNI atlas can be obtained by composing its deformation field to the group-mean image (obtained in the first step) and the deformation field from the group-mean image to the MNI atlas image (obtained in the second step). Following the combined deformation pathway, we are able to map all 45×2 labels onto each time-point image. Since temporal coherence is well persevered in the first step, the labeling results across all time-point images are consistent, as shown in Fig. 7. From the second to the fifth columns of Fig. 7, we demonstrate the 4D ROI labeling result on the longitudinal brain images of a normal control subject, along with the MNI brain atlas shown in the first column.

### 7. ROI Analysis

**Figure 8:**
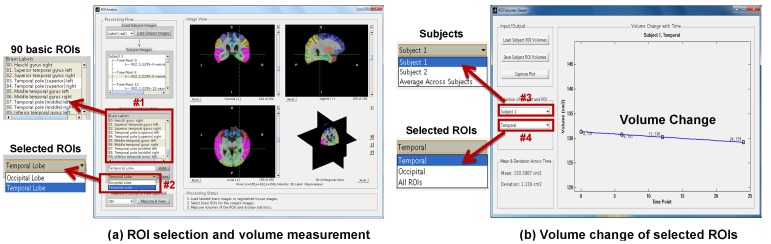
ROI Analysis. (a) The interface for ROI selection and volume measurement. When a brain-extracted image of a subject is selected, the respective labeled ROI map will be overlaid on the brain-extracted image. Then, a set of ROIs (shown in #2 panel) can be created, where each ROI may be a combination of multiple basic ROIs from the 90 basic ROIs (as shown in #1 panel, with Section 2.6 providing the definitions for the 90 basic ROIs). It’s worth noting that the selected basic ROIs in #1 panel are highlighted (in pink) in the labeled ROI map. The volumes of the selected ROIs (in #2 panel) for the longitudinal brain images of all subjects can then be measured automatically and displayed. (b) The interface for display of volume change of selected ROIs. The volume change over time for each ROI (or all ROIs, #4) of each subject (or the average volume across all subjects, #3) can be displayed.

After performing 4D tissue segmentation and 4D brain labeling on longitudinal brain MR images of a group of subjects, we can obtain their respective serial tissue-segmented images and brain-labeled images, as well as their ROI volumes that can be used for longitudinal analysis of ROI volume changes. Specifically, the labeled ROI maps can be overlaid on the respective brain-extracted images (as shown in Fig. 8(a)), where a set of ROIs (such as temporal lobe and hippocampus) can be selected interactively by the user. The volumes of the selected ROIs for the longitudinal brain images of all subjects can then be measured automatically. Finally, the volume change over time for each ROI (or all ROIs) of each subject (or average volume across all subjects) can be displayed in aBEAT. In addition, longitudinal ROI volumes of all subjects can further be exported as a MATLAB ‘.mat’ file for future statistical analysis. Fig. 8(a) shows the interface for ROI selection and volume measurement. Fig. 8(b) shows the interface for display of volume change of selected ROIs.

## Results

The performance of aBEAT in analysis of longitudinal brain MR images is evaluated qualitatively and quantitatively with a large number of longitudinal data from ADNI database. Representative evaluation results for 4D brain extraction, 4D tissue segmentation, 4D brain labeling, and the computation time are presented below.

### 1. 4D Brain Extraction

**Figure 9:**
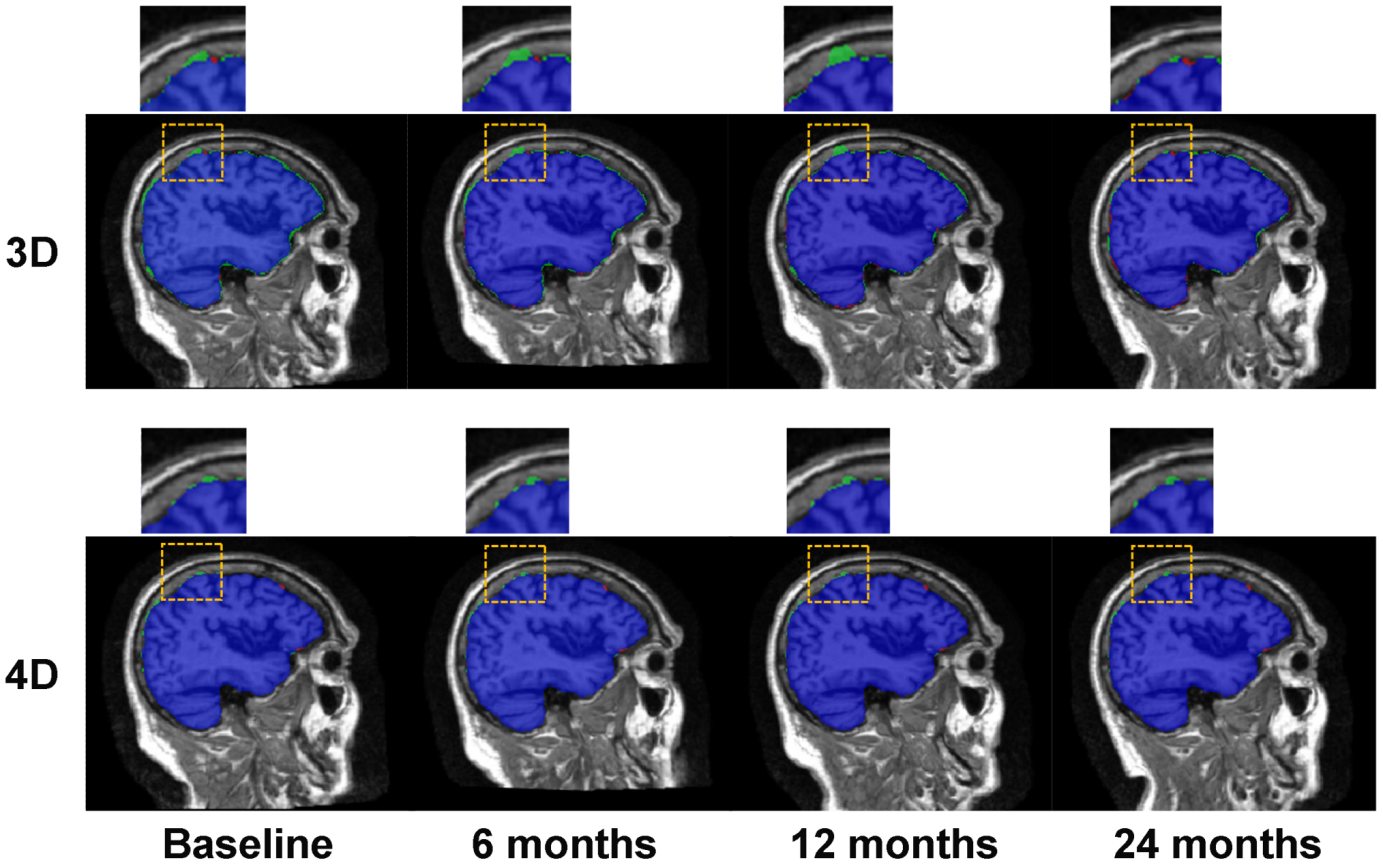
Brain extraction results by the 3D deformable-surface-based method and the 4D method in aBEAT. Sagittal slices are shown. Blue voxels show the common labeling results by automated method and manual ground-truth. Green voxels are the residual non-brain tissues (false positives), and red voxels are the wrongly-removed brain regions (false negatives). The regions in the yellow dotted squares are zoomed, which indicates that the 4D method is more accurate and consistent than the 3D method.

30 subjects (each with 4 time points), including 10 normal controls (NC), 10 mild cognitive impairment (MCI), and 10 Alzheimer’s disease (AD), were employed for the evaluation of 4D brain extraction. The longitudinal brain images of all these subjects were preprocessed (including bias correction and histogram matching) by aBEAT before brain extraction. The brain extraction results by aBEAT were compared with the results obtained by the 3D deformable-surface-based brain extraction method which achieved better performance over the classic BET and BSE methods as shown in [22]. Fig. 9 shows typical brain extraction results by the 3D deformable-surface-based method (top) and aBEAT (bottom), respectively. The small red regions in Fig. 9 indicate false negative voxels (wrongly-removed brain regions w.r.t. manual ground-truth), and the green regions denote false positive voxels (residual non-brain tissues w.r.t. manual ground-truth). Obviously, the 4D brain extraction in aBEAT achieves better performance than the 3D deformable-surface-based method.

Furthermore, the 4D brain extraction was quantitatively evaluated. Specifically, for each time-point image of every subject, the overlap ratios between the manual ground-truth and the automated brain extraction results by the 3D deformable-surface-based method and the 4D method in aBEAT were measured using Jaccard Index, respectively. Notice that the manual ground-truth was semi-automatically delineated (similar to [40]) as follows: automated brain extraction was first performed, followed by manual delineation by experienced human raters using ITK-SNAP [41]. The averaged Jaccard Index degrees (across all subjects and all time points) for the 3D deformable-surface-based method and aBEAT are 0.96±0.02 and 0.98±0.005, respectively, which quantitatively indicates better performance achieved by the 4D brain extraction in aBEAT.

### 2. 4D Tissue Segmentation

**Figure 10:**
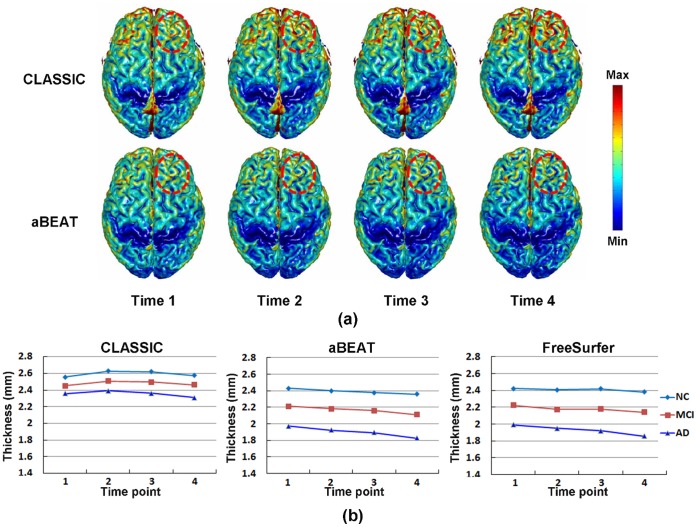
Tissue segmentation results. (a) Cortical thickness maps derived by CLASSIC (the upper row) and aBEAT (the lower row) from a normal control subject. Circles indicate the region with dramatic thickness changes by CLASSIC, while consistent measurement achieved by aBEAT. (b) Changes of mean cortical thickness derived by CLASSIC (left), aBEAT (middle), and FreeSurfer (right) for the NC, MCI, and AD groups, respectively.

Ninety subjects (each with 4 time points in 24 months), including 30 NC, 30 MCI, and 30 AD, were employed for the evaluation of 4D tissue segmentation in aBEAT (after brain extraction). To demonstrate the advantage of aBEAT in 4D tissue segmentation, we compared its results with those obtained using CLASSIC [25]. Specifically, cortical thickness maps were constructed from the tissue-segmented images generated by CLASSIC and aBEAT, respectively. For each cortical thickness map, the cortical surface was reconstructed using the function “isosurface” in MATLAB, while the cortical thicknesses at surface vertices were colored using the function “isocolors” in MATLAB. Typical cortical thickness maps (which were obtained from a NC subject) by the two methods are shown in Fig. 10(a). We can see clearly, e.g., in the frontal lobe, that the cortical thickness by CLASSIC changes dramatically over time, while it is much consistent by aBEAT.

Furthermore, we measured the average cortical-thickness changes for each group (i.e., NC, MCI, and AD), using the cortical-thickness maps derived by CLASSIC and aBEAT, respectively. Specifically, we first calculated the mean cortical thickness for each time-point image of each subject, and then averaged the longitudinal mean cortical thicknesses from all subjects in each group. In Fig. 10(b), we show the longitudinal changes of mean cortical thickness obtained by CLASSIC and aBEAT. As we can see, the mean cortical thickness by aBEAT declines obviously and smoothly along time, while not obviously by CLASSIC. The lowest mean cortical thickness and the largest decrease of mean cortical thickness are both coming from AD group, which agrees with previous findings in the literature [42], [43]. Besides, we also measured the longitudinal changes of mean cortical thickness of each group using the longitudinal processing pipeline recently included in FreeSurfer [44]. It can be seen that the curves by aBEAT are smoother than those by FreeSurfer, especially for the NC and MCI groups.

### 3. 4D Brain Labeling

**Figure 11:**
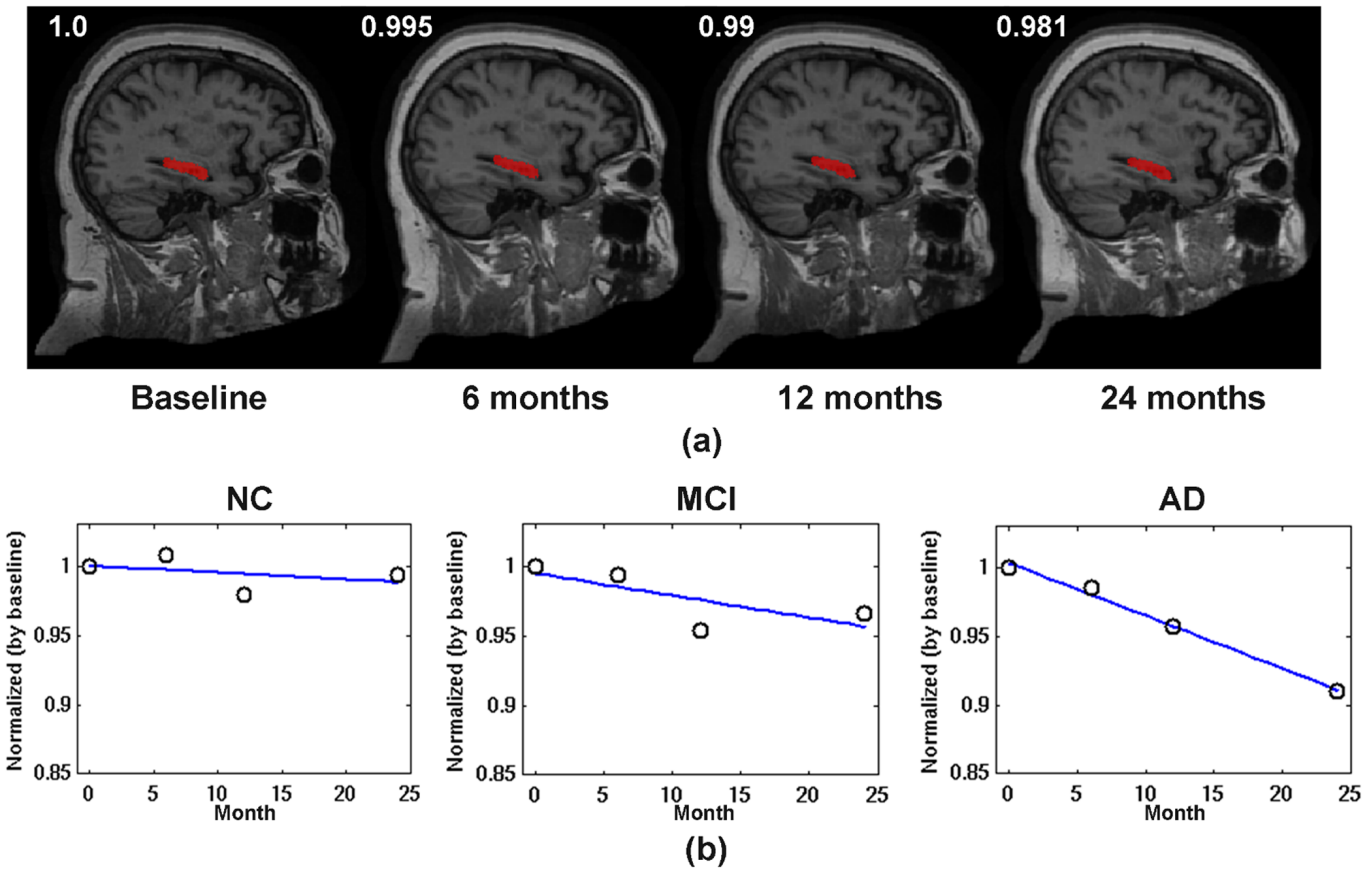
Brain labeling results. (a) Automated 4D labeling results of hippocampus (in red) for a typical normal control subject, with four example slices provided. The hippocampal volume, which was normalized by baseline, decreases slightly from 1 (baseline) to 0.995 (6 months), 0.99 (12 months), and 0.981 (24 months). (b) The temporal development trends of hippocampal GM volume (also normalized by baseline) for the NC, MCI, and AD groups, respectively. The blue line in each plot is a linear fitting for the mean hippocampal GM measured at different time-points.

Fifteen subjects (5 NC, 5 MCI, and 5 AD, each with 4 time points at baseline, 6^th^, 12^th^, and 24^th^ months) were evaluated for automatic 4D ROI labeling using aBEAT. For longitudinal images of each subject, the WM, GM, and CSF were first segmented from the brain-extracted images using the 4D tissue segmentation module as evaluated above. Then, the ROIs in the MNI atlas were simultaneously mapped onto each time-point image to obtain the labeling maps by the 4D brain labeling module in aBEAT. Fig. 11(a) shows the automatically-labeled hippocampus (in red) on the sagittal view for a typical normal control subject. We can see that the hippocampus was accurately and consistently labeled at different time-points. To sensitively detect small neuronal changes in hippocampus [45], the hippocampal GM at each time-point image of each subject was further obtained, by masking the hippocampus ROI label with the GM map obtained from tissue segmentation result. The temporal change trends of hippocampal GM volume (normalized by the volume at baseline) are illustrated in Fig. 11(b) for all groups (NC, MCI, and AD). Notice that, for each group, the temporal change trend was estimated from the average change of hippocampal GM volume across all subjects in the group. It can be seen that, the decrease of hippocampal GM is subtle for NC, while very obvious for MCI and AD. The AD group shows the largest hippocampal GM reduction. These results are in agreement with previous findings by Kitayama et al. [45], Chetelat et al. [46], Colliot et al. [47], and Schuff et al. [9].

### 4. Computation Time

The computation time of aBEAT was estimated on a longitudinal dataset with 4 time points (i.e., 4 images were acquired for one subject at 4 different time points) and also on a cross-sectional dataset with 4 single-time-point images (i.e., 4 images were acquired from 4 different subjects at certain time point), respectively. The voxel size and volume size of each image are 1×1×1 mm^3^ and 256×256×256, respectively. Experiments were performed on a server with 8 CPU cores (Intel Xeon, 2.4 GHz) in Linux operating system. The total memory size of the server is 16 GB. The longitudinal dataset and cross-sectional dataset independently underwent the analysis pipeline in aBEAT (from image preprocessing to brain labeling as shown in Fig. 1), referred to as 4D analysis and 3D analysis, respectively. The computation time taken in each step of the 4D or 3D analysis is given in Table 1. The overall processing times for the 4D analysis and 3D analysis were 6.7 hours and 2.3 hours, respectively. The 4D analysis took more time, as it had to process all longitudinal images using just one thread, while the 3D analysis used multiple threads to process the cross-sectional images parallelly. In the future, we will accelerate the 4D/3D analysis in aBEAT by using more advanced technology, such as parallel computing based on Graphics Processing Units (GPU).

**Table 1:** Computation time taken in each major module for the 4 longitudinal or cross-sectional images.

	Image Preprocessing	Brain Extraction withCerebellum Removal	Tissue Segmentation	Brain Labeling
**4D Analysis (Longitudinal)**	1.64 Minutes	18.4 Minutes	4 Hours	2.38 Hours
**3D Analysis (Cross-sectional)**	1.43 Minutes	16.2 Minutes	1.15 Hour	0.85 Hours

## Conclusion and Discussion

We have developed the aBEAT software with GUIs for 4D analysis of longitudinal brain MR images. The most significant feature of the aBEAT software is that it integrates a group of 4D image analysis algorithms and further provides a user-friendly platform for various 4D brain image analysis tasks, such as brain tissue segmentation and ROI labeling. Specifically, the integration of the advanced 4D brain extraction, 4D tissue segmentation, and 4D brain labeling algorithms ensures accurate and consistent measurement and analysis of longitudinal brain MR images. In addition, aBEAT can also be applied to 3D images for cross-sectional studies, i.e., by using a 3D deformable-surface-based method for brain extraction [22], a coupled level-sets algorithm for 3D tissue segmentation [26], and a symmetric diffeomorphic registration method for 3D brain labeling [30]). So far, a Linux-based standalone software package for aBEAT has been released on the website of Neuroimaging Informatics Tools and Resources Clearinghouse (NITRC). A computer with 8 GB memory (or more) is recommended for analysis of longitudinal images using the software package.

The five major modules in aBEAT (as shown in Fig. 1) interact with each other seamlessly as explained below. The image preprocessing module corrects bias field in the intensities of input images and further normalizes them to match with those in the baseline image, thus benefiting the subsequent processing steps. The brain extraction module removes non-brain tissues and produces brain-extracted images, which facilitates the segmentation of WM, GM, and CSF by the tissue segmentation module. The brain-extracted images and the tissue-segmented images are then used in the brain labeling module for labeling brain ROIs, which can be analyzed statistically in the ROI analysis module. These five modules work sequentially for completing the processing and analysis of brain images. Importantly, each module can also perform its respective task independently, e.g., performing brain extraction by using only the brain extraction module.

aBEAT can be applied to many medical studies. For example, we can use it to segment brain tissues (i.e., GM, WM, and CSF) and brain ROIs (i.e., hippocampus) from longitudinal brain MR images of a subject, and then analyze temporal changes of brain tissues and ROIs to determine whether the subject has certain brain disease such as AD [2] or schizophrenia [3]. We can also use it for analysis of cross-sectional brain MR images, i.e., classifying the subjects into different groups (e.g., with high-risk psychosis or not [48]) according to the measured brain tissues and ROI labels. In addition to these examples on volume-based analysis, the brain tissues and ROI labels obtained by aBEAT can further be used for cortical surface reconstruction and the analysis of cortical ROIs [43].

Our current software package has several limitations, which also indicates the future direction of our work. (1) Although the volume-based ROI analysis function is available in aBEAT, surface-based ROI analysis function is not included yet. Therefore, 4D/3D surface reconstruction tools [43] are still required to reconstruct cortical surfaces from the segmented brain tissue maps. In addition, the measurement tools (i.e., cortical thickness estimator) and visualization tools (i.e., for rendering cortical-thickness map) [43] are also required. We will integrate these tools in our future version of the aBEAT software. (2) The parallel computing strategy used in aBEAT (as described in Section 2.2) can take advantage of multiple processor cores to accelerate image analysis. However, as tissue segmentation was implemented in MATLAB (not as efficient as C/C++ languages) and brain labeling was not fully parallelized, the computational speed of aBEAT is still limited. In the future, we will use C/C++ and GPGPU (General-Purpose computation on Graphics Processing Units) to speedup these algorithms and thus make our software computationally more efficient. (3) Currently, only the Linux version of our software is available. In the future, we will make cross-platform software for aBEAT. (4) Although the Analyze file format is one of the most popular file formats, it is currently the only file format supported by aBEAT. Therefore, users have to use other programs to convert the image file formats, e.g., between DICOM and Analyze formats. In the future, we will support more file formats to ease use of our software. (5) Currently, aBEAT is used for analysis of MR T1 images (the most widely-used type of MR images for adult brain). In the future, we will extend the software for analysis of other types of MR images such as T2 and FA images.

aBEAT is a free software for academic use. The Linux-based standalone software package and tutorial are available at http://www.nitrc.org/projects/abeat. For convenience of using this software, two NC datasets (each with 4 time points) from ADNI database are included in the package. The tutorial describes how to install and use aBEAT software correctly. In addition, frequently asked questions (FAQ) from users and the answers are also provided with the tutorial to address possible questions that new users may have during the use of this software package. User feedbacks are greatly welcomed for further improvement of this software package.
